# Multiple Gastric Erosion Early after a 3 V Lithium Battery (CR2025) Ingestion in an 18-Month-Old Male Patient: Consideration about the Proper Time of Intervention

**DOI:** 10.1155/2016/3965393

**Published:** 2016-11-07

**Authors:** Ioannis Patoulias, Christos Kaselas, Dimitrios Patoulias, Konstantinos Farmakis, Eleni Papacrivou, Maria Kalogirou, Thomas Feidantsis

**Affiliations:** 1st Pediatric Surgery Clinic, Aristotle University of Thessaloniki, G. H. G. Gennimatas, 41 Ethnikis Aminis Street, 54635 Thessaloniki, Greece

## Abstract

*Introduction*. Button battery ingestion is considered an emergency situation in pediatric patients that needs to be managed promptly; otherwise, it may lead to serious and potentially lethal complications, especially when it is impacted in the esophagus. Less attention has been given in cases where the battery passes into the stomach, with guidelines for emergency intervention being based on the presence of symptoms.* Case Report*. We present a case of an 18-month-old male patient who presented to our emergency department after button battery ingestion. He did not have any symptoms and no pathological findings were encountered during clinical examination. X-ray investigation revealed the presence of the battery in the stomach. The patient was admitted for observation and two hours later he had two episodes of vomiting. He underwent urgent endoscopic removal of the battery where multiple acute gastric mucosal erosion in place of direct contact of the battery's negative pole with the mucosa of the gastric antrum was found.* Conclusion*. In specific cases the urgent endoscopic intervention for removal of an ingested button battery that is located in the stomach even in asymptomatic patients should be suggested.

## 1. Introduction

Button battery ingestion is a serious problem that health providers need to manage. The frequency of the phenomenon is increasing, especially in small children as they gain more access to electric toys and devices [1]. Impaction of the battery in the esophagus poses the greatest danger as it is correlated with serious complications, occasionally lethal if left untreated [2, 3]. Specific guidelines have been issued on when, where, and how to treat such cases [3].

On the other hand, in cases where the button battery passes the lower esophageal sphincter and is found in the stomach, management guidelines are not so clear, relying only on the presence of symptoms in order to decide whether an endoscopic removal should be performed [2, 3].

We present a case of an 18-month-old male patient who ingested a button battery and was found to have multiple superficial and deep erosion of the gastric mucosa after the battery was removed from the stomach endoscopically 4 hours later. We present our concerns and debate on possible alternative approaches on that matter.

## 2. Case Report

An otherwise healthy 18-month-old boy was brought in the emergency department by his parents due to possible button battery ingestion. The parents suggested that the incident took place at their home no more than half an hour prior to their arrival. The child was asymptomatic and alert. He did not show any signs of discomfort, dyspnea drooling, or pain.

Clinical evaluation did not reveal anything significant. Vital signs were normal and oxygen saturation was >96%. Evaluation of the oral cavity did not show any mucosal damage or any other signs of ingestion of a foreign body. Auscultation of the lungs was normal and the abdomen was soft at palpation with no signs of distention or tenderness.

The child was immediately referred for a radiographic evaluation. A foreign body located in the stomach that simulated a button battery or a coin was found in the anteroposterior X-ray. Immediately, a second lateral X-ray was taken that revealed the characteristic “step-off” sign, indicative of a button battery. It is created due to the different diameters on the flat and convex sides of the battery.

At that time the father arrived to the emergency department and supplied an identical battery to the one that was supposedly ingested by the child. This was a 20 mm diameter 3 V lithium battery (CR2025). The increased diameter of the battery and with the 3 V current that it creates led us to the decision to hospitalize the child and follow up his condition.

He was put in “nil per os” diet and intravenous hydration. At the second hour of hospitalization the child had two consecutive vomiting episodes without showing any discomfort or change to his mood or clinical condition thereafter. Despite the above, we decided to change our approach and a gastroscopy was scheduled.

**Figure 1:**
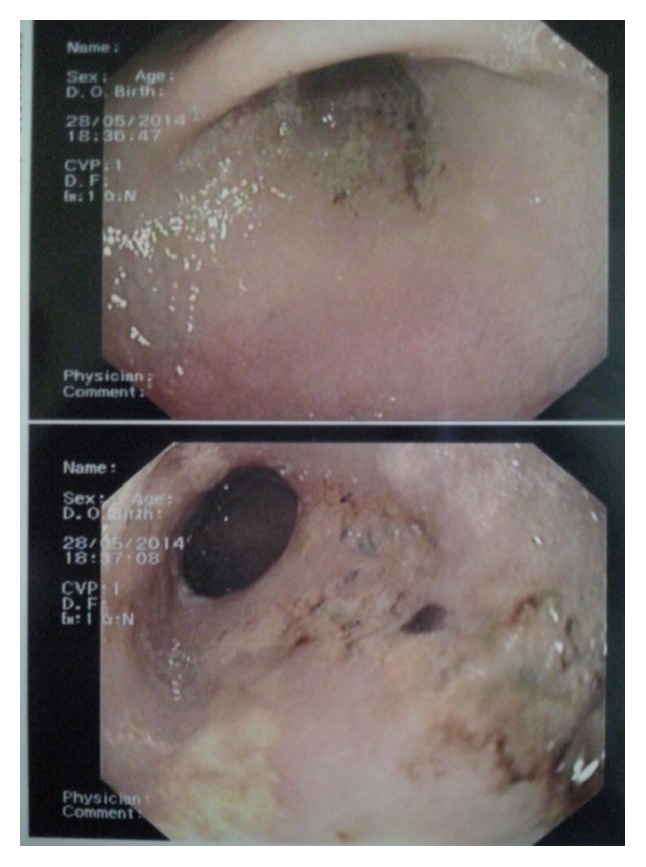
Button battery in the stomach. Note the multiple mucosal erosion.

During endoscopy, the battery was found in the antrum alongside the greater curvature of the stomach, with its negative pole in contact with the gastric mucosa, and was removed in a basket (Figure 1).

**Figure 2:**
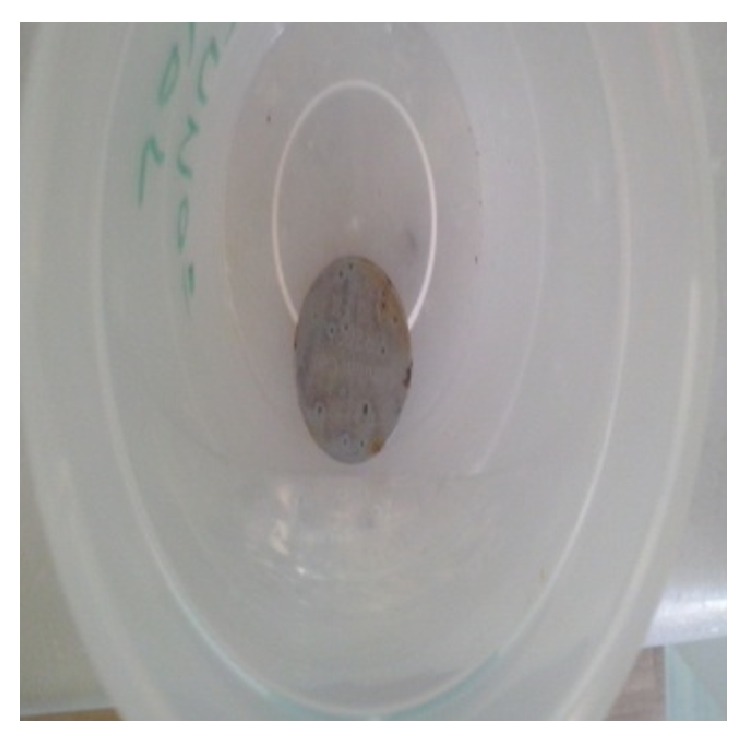
Withdrawn specimen. Note the significant corrosion of its surface.

A thorough inspection of the area revealed multiple superficial and deep erosion of the mucosa. Upon completion, we examined the battery itself and we found that the battery was significantly corroded (Figure 2).

The patient's postoperative period was uneventful and he was discharged on the second postoperative day. Follow-up 15 days and 2 months after the event was normal.

## 3. Discussion

Our case describes the presence of multiple acute gastric mucosal erosion found during endoscopy in an 18-month-old male patient, 4 hours after CR2025 3 V button battery ingestion. Our approach and management plan was to hospitalize the patient due to his small age in relation to the large diameter and high voltage of the battery. The evolution of the patient's clinical condition and the unexpected findings during endoscopy seem to have justified our decision and maybe suggest that in certain cases such an approach or even more aggressive one can be warranted.

The incidence of button battery ingestion is rising, possibly due to increased access of children to electric toys and other household devices [1, 2]. In a large series of the last 20 years, it is postulated that 68.1% of button battery ingestion incidents occurred in children younger than 6 years old [3]. The same report underlined the increased usage of high voltage (3 V) and large diameter (>20 mm) lithium batteries, possibly due to the advantages of such a cell, mostly its electrochemical efficiency, high energy density, long shelf life, and cold tolerance. This is inevitably linked to the increased incidence of large diameter batteries ingestion (from 1% to 18%) and especially those containing lithium (from 1.3% to 24%) [3].

27% of children developed serious complications, while in 54% of those who died, delay in diagnosis and thus in treatment was noticed [3]. This is attributed to the fact that the children do not have well-developed speaking skills but also to the absence of typical symptoms. Pediatric patient is symptomatic either when the ingested battery causes impaction in the esophagus or when it causes acute gastric mucosal erosion [3].

The risk of an ingested button battery depends on the site of impaction, its chemical consistency, its diameter (especially ≥20 mm) and voltage (≥3 V), the age of the child, and time to diagnosis and intervention [4]. It is reported that 12.6% of children <6 years old that ingested a large diameter button battery (20–25 mm), which was impacted in the esophagus, developed serious or lethal complications [5]. Factors associated with clinical significant outcome included cell diameter 20–25 mm, age <4 years, and ingestion of >1 batteries [3, 5].

Specific guidelines exist for esophageal button battery impaction, proposing immediate emergency endoscopic removal [3, 6]. When the battery is located in the stomach, it is proposed that a more conservative approach should be adopted. More specifically, current guidelines for asymptomatic patients indicate that when the patient is <6 years old and battery diameter is >15 mm, inspection of the child's stools and repetition of the X-ray after 4 days should be performed, in case the battery is not passed with stools [2, 3, 6]. Intervention is proposed if symptoms emerge or the battery is still located in the stomach. In a recent article, Amanatidou et al. do not take into account patient's age or the battery's diameter and propose outpatient management of asymptomatic patients and radiologic reevaluation on the 6th day after the incident [7].

In our case a 20 mm 3 V lithium battery was ingested. Even though our patient was initially asymptomatic and the battery was lying in the stomach, we decided to keep the patient for observation in the hospital. Looking back, we are concerned whether the first or second episode of vomiting would be a strong stimulus to alert the parents, as such episodes can be anticipated in an 18-month-old baby.

The major injury mechanism after lithium button battery ingestion is the generation of external current, electrolysis of tissue or mucosal fluids, and local generation of hydroxide [3, 8]. Direct contact of the lower pole of the battery with the gastric mucosa induces local generation of hydroxide, which is strongly erosive [3, 4]. Leakage does not cause significant local injury as lithium button batteries do not contain an alkaline electrolyte but rather a mildly irritating organic electrolyte [3]. Furthermore, progression of the injury may continue and spread even after removal of the battery, due to remaining hydroxide or induced tissue weakness in the place of erosion [3, 4]. In our case we consider that the battery's 3 V voltage created a strong external current that led to extensive injury of the patient's immature gastric mucosa.

Takagaki et al. faced a similar problem. They managed a 15-month-old patient that had ingested 3 button batteries that were lying in the stomach. They too decide to perform a more aggressive approach and proceeded to endoscopic removal of the batteries although symptoms were minor just to find extensive erosion and necrotic gastric mucosa [9].

Honda et al. had to proceed to a laparotomy in order to remove a button battery from the stomach and to treat a 3-month-old infant that had ingested it two days ago but his clinical condition was severely deteriorated [10].

Our case among others indicates that guidelines regarding management of button batteries ingestion in infants and toddlers should be further evaluated, especially in cases that these are located in the stomach and the patients remain asymptomatic. We conclude the following.

- Urgent endoscopic removal of the ingested coin battery depicted in the stomach should be conducted in the presence of the following prerequisites:
  1. diameter > 15 mm,
  2. lithium battery,
  3. age < 4 years,
  4. presence of symptoms,
  5. >1 ingested batteries,
  6. magnet ingestion at the same time.
- We consider the appliance of the updated guidelines of the National Capital Poison Center as sufficient in those cases, in which the above criteria (A) are not met.

## References

[1] Marom T., Goldfarb A., Russo E., Roth Y. (2010). Battery ingestion in children. *International Journal of Pediatric Otorhinolaryngology*.

[2] McConnell M. K. (2013). When button batteries become breakfast: the hidden dangers of button battery ingestion. *Journal of Pediatric Nursing*.

[3] Litovitz T., Whitaker N., Clark L., White N. C., Marsolek M. (2010). Emerging battery-ingestion hazard: clinical implications. *Pediatrics*.

[4] Hamilton J. M., Schraff S. A., Notrica D. M. (2009). Severe injuries from coin cell battery ingestions: 2 case reports. *Journal of Pediatric Surgery*.

[5] Litovitz T., Whitaker N., Clark L. (2010). Preventing battery ingestions: an analysis of 8648 cases. *Pediatrics*.

[6] Jatana K. R., Litovitz T., Reilly J. S., Koltai P. J., Rider G., Jacobs I. N. (2013). Pediatric button battery injuries: 2013 task force update. *International Journal of Pediatric Otorhinolaryngology*.

[7] Amanatidou V., Sofidiotou V., Fountas K. (2011). Button battery ingestion: the Greek experience and review of the literature. *Pediatric Emergency Care*.

[8] Kay M., Wyllie R. (2013). Foreign body ingestions in the pediatric population and techniques of endoscopic removal. *Techniques in Gastrointestinal Endoscopy*.

[9] Takagaki K., Perito E. R., Jose F. A., Heyman M. B. (2011). Gastric mucosal damage from ingestion of 3 button cell batteries. *Journal of Pediatric Gastroenterology and Nutrition*.

[10] Honda S., Shinkai M., Usui Y. (2010). Severe gastric damage caused by button battery ingestion in a 3-month-old infant. *Journal of Pediatric Surgery*.

